# Supplementary material for: "Knowledge regarding cardiopulmonary resuscitation among health assistants in Nepal: A cross-sectional study"
Source: PLoS One. 2023 Nov 9;18(11):e0293323. doi: 10.1371/journal.pone.0293323 (PMC10635484; doi:10.1371/journal.pone.0293323)
Supplement: S1 File — (DOC) [file pone.0293323.s001.doc]

Reliability


Scale: ALL VARIABLES


Case Processing Summary	
	N	%	
Cases	Valid	51	100.0	
	Excludeda	0	.0	
	Total	51	100.0	

a. Listwise deletion based on all variables in the procedure.	


Reliability Statistics	
Cronbach's Alpha	N of Items	
.683	17	


Reliability


Notes	
Output Created	03-SEP-2023 15:35:10	
Comments		
Input	Data	C:\Users\pathiyilravi\OneDrive - International Medical University\MyPC\Manuscripts\Bivek CPR\Pilot study.sav	
	Active Dataset	DataSet1	
	Filter	<none>	
	Weight	<none>	
	Split File	<none>	
	N of Rows in Working Data File	51	
	Matrix Input		
Missing Value Handling	Definition of Missing	User-defined missing values are treated as missing.	
	Cases Used	Statistics are based on all cases with valid data for all variables in the procedure.	
Syntax	RELIABILITY
  /VARIABLES=One Two Three Four Five Six Seven Eight Nine Ten Eleven Twelve Thirteen Fourteen
    Fifteen Sixteen Seventeen
  /SCALE('ALL VARIABLES') ALL
  /MODEL=ALPHA
  /SUMMARY=TOTAL.	
Resources	Processor Time	00:00:00.00	
	Elapsed Time	00:00:00.01	


Scale: ALL VARIABLES


Case Processing Summary	
	N	%	
Cases	Valid	51	100.0	
	Excludeda	0	.0	
	Total	51	100.0	

a. Listwise deletion based on all variables in the procedure.	


Reliability Statistics	
Cronbach's Alpha	N of Items	
.683	17	


Item-Total Statistics	
	Scale Mean if Item Deleted	Scale Variance if Item Deleted	Corrected Item-Total Correlation	Cronbach's Alpha if Item Deleted	
One	8.67	8.547	.221	.677	
Two	8.80	8.041	.248	.673	
Three	9.18	7.668	.330	.662	
Four	8.90	7.530	.447	.648	
Five	9.06	7.976	.217	.678	
Six	8.71	8.012	.490	.656	
Seven	8.90	7.530	.447	.648	
Eight	9.43	8.130	.239	.673	
Nine	9.47	8.694	.004	.696	
Ten	9.04	7.838	.271	.670	
Eleven	9.02	7.500	.406	.652	
Twelve	9.39	8.243	.167	.682	
Thirteen	9.16	7.815	.273	.670	
Fourteen	8.88	7.426	.508	.641	
Fifteen	8.90	8.210	.165	.683	
Sixteen	9.53	8.854	-.059	.697	
Seventeen	9.00	7.720	.324	.663	
