# Supplementary material for: "Knowledge regarding cardiopulmonary resuscitation among health assistants in Nepal: A cross-sectional study"
Source: PLoS One. 2023 Nov 9;18(11):e0293323. doi: 10.1371/journal.pone.0293323 (PMC10635484; doi:10.1371/journal.pone.0293323)
Supplement: S2 File — (DOCX) [file pone.0293323.s002.docx]

**Questionnaire**

**Knowledge regarding cardiopulmonary resuscitation (CPR) among health assistants in Nepal: a cross-sectional study.**

**Section I: Demographics**

1. Age (in years):
2. Province:
3. District:
4. Where do you work?
5. Government hospital
6. Private clinics/hospitals
7. Semi-government
8. Not working
9. Others: Specify……………………..

If it is a government hospital, mention the category

1. Primary level (Health Post, Primary Health Center)
2. Secondary level (district hospitals or provincial hospitals)
3. Tertiary level (federal hospitals) (This can be removed)
4. Gender:
5. Male
6. Female
7. Others
8. Currently, your average monthly income in (NPR) is ……………………
9. How many years of work experience do you have after PCL (General Medicine) ? …………………

**Section II**

**CPR (Cardio Pulmonary Resuscitation)**

1. Have you heard about CPR (Cardio Pulmonary Resuscitation)?
2. Yes
3. No
4. Have you taken any training in CPR (Cardio Pulmonary Resuscitation)?
5. Yes
6. No

If yes,

How many hours /days of training have you taken?

What was the name of the training provider institution/hospital?

1. Have you ever performed CPR (Cardio Pulmonary Resuscitation)?
2. Yes
3. No

If yes, how many times have you performed ?........

1. Did you attend any theory classes on CPR (Cardio Pulmonary Resuscitation) during your 3 years of Health assistant training?
2. Yes
3. No
4. Don’t remember

If yes, mention the duration attended in hours, …………….

1. Did you receive any practical practice sessions on CPR (Cardio Pulmonary Resuscitation) during your 3 years of Health assistant training?
2. Yes
3. No

If yes, mention the duration attended in hours, …………….

1. Did you receive any practical practice session on CPR (Cardio Pulmonary Resuscitation) from the hospital / health post where you are currently working?
2. Yes
3. No

If yes, mention the duration attended in hours, …………….

1. Do you know the difference between adult CPR and child CPR?
2. Yes
3. No

If yes, from where did you know it from?

1. Course
2. Self-education
3. Colleagues

If yes, what is the compression depth in CPR in Adults, Children, and Infant

Adults (cm)...........

Children (cm)........

Infant (cm)...... (Make each one a separate question)

**BLS (Basic Life Support)**

1. Have you heard about BLS (Basic Life Support)?
2. Yes
3. No
4. Have you taken any training in BLS (Basic Life Support)?
5. Yes
6. No

If yes,

How many hours of training have you taken?

What was the name of the training provider institution/hospital?

Was the training certified by the American Heart Association? (Yes/No) (The respondent answers this only if s/he mentions yes to BLS training)

**Advanced Cardiac Life support**

1. Have you heard about ACLS (Advanced Cardiac Life Support)?
2. Yes
3. No
4. Have you taken any training in ACLS (Advanced Cardiac Life Support)?
5. Yes
6. No

If yes,

How many hours /days of training have you taken?

What was the name of the training provider institution/hospital?

Was the training certified by American Heart Association? (Yes/No) (The respondent answers this only if s/he mentions yes to BLS training)

1. Do you know to interpret an ECG (Electro Cardio Gram)? (Is this something an HA is expected to do? If not we can remove)
2. Yes
3. No
4. Have you ever heard of AED (Automated External Defibrillator)?
5. Yes
6. No
7. Have you ever used a defibrillator?
8. Yes
9. No

If yes, how many times: ……………………..

1. Have you ever heard of shockable and non-shockable rhythm?
2. Yes
3. No

**Section III: Knowledge of Cardio Pulmonary Resuscitation**

|  |  | Agree | Disagree | Don’t know |
| --- | --- | --- | --- | --- |
|  |  |  |  |  |
| 1 | Cardio Pulmonary Resuscitation is an emergency procedure to save life |  |  |  |
| 2 | Only doctors can perform CPR  If not, who else do you think can do CPR? (Let us remove this may lead respondents to the answer) |  |  |  |
| 3 | CPR is only done for myocardial infarction |  |  |  |
| 4 | CPR is done for cardiac arrest |  |  |  |
| 5 | CPR done 10 minutes after cardiac arrest has a low survival chance |  |  |  |
| 6 | The compression ratio is 2 rescuer adult CPR is 30:2 |  |  |  |
| 7 | CPR is only done in a hospital setting |  |  |  |
| 8 | The AHA follows the acronym A-B-C during the CPR  A: Airway  B: Breathing  C: Chest compression |  |  |  |
| 9 | We should tap the hand to check the person’s responsiveness |  |  |  |
| 10 | The compression is done with the heel of the dominant hand |  |  |  |
| 11 | For quality CPR, 5 cycles should be performed in 2 minutes |  |  |  |
| 12 | We should not allow the chest to recoil during compression |  |  |  |
| 13 | If there is pulse but no breathing, do not continue compression |  |  |  |
| 14 | The depth of the quality CPR in adult is at least 2 inch (5 cm) |  |  |  |
| 15 | The compression rate of the quality CPR in adult is 100-120 compression per minute |  |  |  |
| 16 | The **first step** in AHA chain of survival for adult in In-Hospital Cardiac Arrest (IHCA) is high quality CPR. |  |  |  |
| 17 | Adult cardiac arrest algorithm recommends 1 breath every 6 seconds under advanced airway |  |  |  |

A: Airway

AED: Automated External Defibrillator

AHA: American Heart Association

B: Breathing

BLS: Basic Life Support

C: Chest compression

CPR: Cardio Pulmonary Resuscitation

ECG: Electro Cardio Gram

**Note: Lines with yellow highlights have been modified or removed in the final questionnaire.**
