# Supplementary material for: "Knowledge regarding cardiopulmonary resuscitation among health assistants in Nepal: A cross-sectional study"
Source: PLoS One. 2023 Nov 9;18(11):e0293323. doi: 10.1371/journal.pone.0293323 (PMC10635484; doi:10.1371/journal.pone.0293323)
Supplement: Attachment — Submitted filename: Revision letter.docx [file pone.0293323.s004.docx]

4^th^ October 2023

To

The Editor

PLoS One

Sub: Submission of the revised version of manuscript PONE-D-23-18050R

Dear Editor

We are submitting the revised version of the manuscript PONE-D-23-18050R. The manuscript has been revised and the references have been reformatted.

The response to specific comments follows.

Please review your reference list to ensure that it is complete and correct. If you have cited papers that have been retracted, please include the rationale for doing so in the manuscript text or remove these references and replace them with relevant current references. Any changes to the reference list should be mentioned in the rebuttal letter that accompanies your revised manuscript. If you need to cite a retracted article, indicate the article’s retracted status in the References list and also include a citation and full reference for the retraction notice.

*We have edited the references as suggested.*

Additional Editor Comments:

Majority of comments from reviewers in earlier reviews well addressed however there is a comment by Reviewer 1 to address in introduction section and a comment regarding references need to be addressed.

Also, overall referencing is not alligned to PLOS guideline "References are listed at the end of the manuscript and numbered in the order that they appear in the text. In the text, cite the reference number in square brackets (e.g., “We used the techniques developed by our colleagues [19] to analyze the data”). PLOS uses the numbered citation (citation-sequence) method and first six authors, et al." Please kindly revise throughly considering the PLOS authors guideline cheslist <https://journals.plos.org/plosone/s/submission-guidelines>.

*We have revised the reference formatting and citation in the text.*

Reviewers' comments:

Reviewer #1: 1. Please omit this sentence from the introduction section "The null hypothesis proposes that there is no significant
100 difference in knowledge scores according to respondents’ demographic and other characteristics."

*The sentence has been omitted.*

2. Please make sure that reference 13 is correct in line with Vancouver style

*The reference has been edited.*

Reviewer #2: Thank you for re-inviting me to review the manuscript. After going through the author's response to the reviewer comments on the revised manuscript submitted to PLOS ONE, the authors have done a thorough job of addressing the major concerns raised during the initial review process. Here are my thoughts on some key points:

Sample Size and Recruitment:
- The authors have provided more details on participant recruitment, including keeping the survey open to allow more participants from remote areas. They have justified the larger final sample size.
*Thank you.*
Questionnaire Distribution and Consent:
- The process of online questionnaire distribution via social media and obtaining informed consent is now clearly described.
*Thank you.*

Questionnaire Validity and Pretesting:
- The authors have clarified that pilot study participants were excluded from the final analysis. Details are provided on content validation and questionnaire refinement based on expert input. The supplementary material with the questionnaire and Cronbach's alpha calculations enhances transparency.
*Thank you.*

Results:
- The sample size discrepancy between methods and results has been addressed. The authors clearly state pilot study participants were not included in the final analysis.
*Thank you.*

Discussion:
- Specific, practical recommendations for improving CPR training for health assistants have now been included, enhancing the manuscript's relevance.
*Thank you.*

Overall, the authors have been responsive to the major critiques and suggestions. The revised manuscript reads much stronger in terms of methodology and transparency. The additional details provided instill greater confidence in the validity of the study and its conclusions. I would recommend accepting this revised manuscript for publication in PLOS ONE, pending any final minor edits during the production process. The authors have undertaken considerable effort to improve the manuscript quality based on the reviewer feedback.

*Thank you.*

The changes have been carried out using track changes.

Hoping for a favorable consideration

With regards

Dr Singh

Dr Shankar
